# Management and investigation of neonatal encephalopathy: 2017 update

**DOI:** 10.1136/archdischild-2015-309639

**Published:** 2017-04-06

**Authors:** Kathryn Martinello, Anthony R Hart, Sufin Yap, Subhabrata Mitra, Nicola J Robertson

**Affiliations:** 1 Department of Neonatology, Institute for Women's Health, University College London, UK; 2 Department of Neonatal and Paediatric Neurology, Sheffield Children's Hospital NHS Foundation Trust, Sheffield, UK; 3 Department of Inherited Metabolic Diseases, Sheffield Children's Hospital NHS Foundation Trust, Sheffield, UK

**Keywords:** neonatal encephalopathy, hypoxic ischaemic encephalopathy, neuroprotection, resuscitation of the newborn

## Abstract

This review discusses an approach to determining the cause of neonatal encephalopathy, as well as current evidence on resuscitation and subsequent management of hypoxic-ischaemic encephalopathy (HIE). Encephalopathy in neonates can be due to varied aetiologies in addition to hypoxic-ischaemia. A combination of careful history, examination and the judicious use of investigations can help determine the cause. Over the last 7 years, infants with moderate to severe HIE have benefited from the introduction of routine therapeutic hypothermia; the number needed to treat for an additional beneficial outcome is 7 (95% CI 5 to 10). More recent research has focused on optimal resuscitation practices for babies with cardiorespiratory depression, such as delayed cord clamping after establishment of ventilation and resuscitation in air. Around a quarter of infants with asystole at 10 min after birth who are subsequently cooled have normal outcomes, suggesting that individualised decision making on stopping resuscitation is needed, based on access to intensive treatment unit and early cooling. The full benefit of cooling appears to have been exploited in our current treatment protocols of 72 hours at 33.5°C; deeper and longer cooling showed adverse outcome. The challenge over the next 5–10 years will be to assess which adjunct therapies are safe and optimise hypothermic brain protection in phase I and phase II trials. Optimal care may require tailoring treatments according to gender, genetic risk, injury severity and inflammatory status.

Neonatal encephalopathy (NE) is defined as a condition occurring in babies born over 35 weeks gestational age in which there is disturbed neurological function. The key feature is the disturbance in the degree or quality of consciousness; other features, such as seizures, cardiorespiratory compromise or abnormal tone and reflexes, may occur alongside it but are not necessary to make the diagnosis.^1^ In 2014, the *Task Force on Neonatal Encephalopathy* published new guidelines on criteria for retrospective definition of an intrapartum event sufficient to cause cerebral palsy (CP).^1^ The title of the report was changed from *Neonatal Encephalopathy and Cerebral Palsy* to *Neonatal Encephalopathy and Neurological Outcome* to emphasise that there are many causes of encephalopathy in a newborn baby and that an array of developmental outcomes may arise in addition to CP. Knowledge gaps preclude a definitive test or set of markers that accurately identifies, with high sensitivity and specificity, an infant in whom NE is attributable to an acute intrapartum event. The term NE should be used where no definite aetiological diagnosis is known, and hypoxic-ischaemic encephalopathy (HIE) where clear diagnosis of hypoxia-ischaemia is known to have led to the neonate's clinical state.

## Determining the aetiology of NE

The initial stages of managing NE will be the same for most babies, with good resuscitation and supportive management. However, as the picture evolves and investigations return, clinicians should consider the aetiology of NE as this could lead to specific treatments, aid with prognosis and recurrence risk counselling, and assist with the evaluation of medicolegal implications.

The other aetiologies to consider include:

- Acquired conditions, such as congenital infection, meningitis, haemorrhage, ischaemic or haemorrhagic stroke
- Genetic syndromes or isolated gene conditions
- Neurometabolic disorders, particularly where the stress of delivery leads to decompensation
- ‘Double trouble’ pathologies where a primary pathology leads secondarily to a hypoxic-ischaemic brain injury, like neuromuscular or cardiac disorders
- The neonatal epilepsy syndromes and vitamin responsive seizures
- Non-accidental injury

Assessment should include a detailed history and neonatal examination, possibly parental examination, and the judicious use of investigations.

### History and examination

A history should be obtained from the mother or, if not possible, a review of the medical/antenatal notes or history from the extended family. Salient features to identify are summarised in box 1. The following are recommended: a three-generation family tree, with focus on miscarriages, stillbirths, child or early adult deaths, ‘cerebral palsy’, learning difficulties, seizures, encephalopathy, metabolic conditions and early onset ischaemic strokes.

Box 1 **Features to look out for in history and examination**

Pregnancy/labour history:

- Was there an acute event occurring around the time of birth, such as non-reassuring or abnormal trace on cardiotocograph,^99^ antepartum haemorrhage, placenta previa, cord prolapse?
- Fetal growth on antenatal scans
- Fetal abnormalities on ultrasound scan or antenatal MRI
- Was this a multiple pregnancy (twins, triplets, etc)?
- Maternal infections or carriage of group B Streptococcus
- Maternal hypertension
- Pre-eclampsia
- HELLP syndrome, particularly if associated with acute fatty liver infiltration, may indicate long chain 3 hydroxyacyl-coenzyme A dehydrogenase (LCHAD) deficiency^2^
- Maternal hypotension
- Maternal prescribed drug use
- Maternal illicit drug use, particularly cocaine
- Illness during pregnancy, such as may occur in viral infections that may affect the fetus
- Gestational diabetes
- Trauma, such as accidental falls or road traffic accident, and inflicted (assault)
- Evidence of maternal haemorrhage
- Any predisposing features to a non-accidental injury of baby, if presenting following normal period of consciousness?

*Maternal past medical history*:

- *Multiple miscarriages, stillbirths or neonatal deaths—*consider genetic, thrombophilia and metabolic causes
- *Diabetes:* associated with brain injuries, such as fetal thrombotic vasculopathy and postnatal hypoglycaemia
- *Deep vein thrombosis or other clotting disorders:* suggestive of thrombophilia or clotting disorder and classical homocystinuria
- *Arterial ischaemic stroke:* suggestive of thrombophilia or vascular abnormalities, such as COL4A1 gene mutations
- *Learning difficulties*: suggestive of genetic/metabolic disorder, including myotonic dystrophy which may lead to secondary hypoxic brain injury
- ‘*Family history of cerebral palsy*’*:* suggestive of vascular abnormalities, such as COL4A1 gene mutations, or thrombophilia
- *Cataracts:* may indicate inborn error of metabolism, myotonic dystrophy, COL4A1 mutations
- *Stiffness or startling:* consider myotonic disorders or hyperekplexia
- *Weakness or muscle fatigue:* consider neuromuscular problem like myasthenia gravis or congenital myaesthenic syndrome, especially if ophthalmoplegia or unexplained squint present. If muscle aches, pains and tetany exist, consider maternal hyperparathyroidism
- *Features of autoimmune disorder:* involvement of several endocrine abnormalities, rash or other skin abnormalities like Raynaud's syndrome, eye and kidney abnormalities, muscle aches and pains, heart block
- *Distal weakness of hands or feet, or abnormally shaped toes:* consider peripheral neuropathy

Examination of the parents

This is important where a neuromuscular disorder is suspected.

- Neuropathies—reduced strength distally, suppressed or absent reflexes, abnormally shaped feet/toes, possible loss of sensation in either parent
- Myopathies—proximal weakness, reduced reflexes and normal sensation in either parent
- Neuromuscular junction defects like maternal myasthenia gravis or myaesthenic syndromes—fatigue/weakness on repeated or prolonged testing of grip strength, upward eye gaze or ptosis
- Maternal myotonia in congenital myotonic dystrophy.

Neonatal examination

- Head circumference abnormalities
- Dysmorphic features
- Abnormal fontanelle shape or size
- Features suggestive of a metabolic condition (box 2)^2^
- Rashes suggestive of immune, metabolic conditions or clotting disorders Family History of cerebral palsy:
- External and internal ophthalmoplegia
- Facial weakness
- Features of peripheral involvement, with weakness and reduced reflexes
- Features of spinal involvement—difficult vaginal birth, mixed upper and lower motor neuron finding, sensory level, urinary retention, constipation
- Neonatal hypertonia—while neonates with hypoxic-ischaemic encephalopathy can exhibit hypertonia, tremor, myoclonus and shivering following birth, especially during hypothermia treatment, these usually resolve. A baby who is hypertonic from birth and remains stiff is unlikely to have experienced hypoxic-ischaemic encephalopathy. An approach to the diagnostic evaluation of hypertonic neonates has been proposed previously.2a

A detailed examination of the baby is needed, including a neurological assessment, and repeated neurological examination may be required, as signs can change quickly. Where a neuromuscular disorder is possible, the parents should be examined. Important features to identify in the examination are summarised in box 1, and neonatal features suggestive of a metabolic disorder in box 2.^2^

Box 2 **Features on examination suggestive of metabolic aetiology^2^**

Dysmorphia

- Large fontanelle
- Large, prominent forehead
- Hypertelorism
- Mid-face hypoplasia
- Epicanthic folds
- Flat nasal bridge
- Long philtrum
- Unusual nose, upturned/flared alae nasi
- Ear abnormalities, including low set and external abnormalities to pinna
- Genital abnormalities
- Limb shortening
- Clinodactyly/syndactyly
- Abnormal feet, such as rocker-bottom
- Abnormal, inverted nipples
- Abnormal fat pads

Head size

- Microcephaly
- Macrocephaly

Liver involvement

- Hepatomegaly
- Jaundice

Cardiac

- Failure/cardiomyopathy
- Abnormal ECG

Eye abnormalities

- Cataracts
- Retinitis pigmentosa (noted on ophthalmology review)
- Cherry red spots
- Optic atrophy
- Lens dislocation

Fetal hydrops

### First line investigations

Routine first line bloods are shown in table 1. These basic tests will also allow calculation of the anion gap ((serum sodium+potassium)—(serum bicarbonate+chloride)), with the normal value being <16. Where a clear history of an antenatal /intrapartum event exists, and the clinical presentation, course and first line investigations point towards HIE, no further aetiological investigations are required, although neuroimaging will provide prognostic information.

**Table 1:** Early investigations to assess neonatal encephalopathy

First line investigations	Comment
Full blood count	May suggest infection, haemorrhage, thrombocytopenia.
Clotting	Clotting disorders may be seen in HIE and sepsis, but should also lead the clinician to think about anaemia secondary to inherited coagulation disorders and intracranial haemorrhage.
Direct Coombs test	Evidence of haemolysis.
Liver function test	May be abnormal in HIE but is usually transient unless a severe insult to the liver has occurred. Abnormal liver function tests can be a feature of bilirubin encephalopathy, metabolic conditions, congenital infections, and acute sepsis with bacteria and viruses, including herpes simplex virus.
Urea and electrolytes	May be impaired if the kidneys have had an ischaemic insult but usually improves, unless severe ischaemic injury has occurred. May also be impaired in congenital abnormalities of the kidneys, metabolic conditions.
Whole blood glucose (rather than serum glucose as the latter is around 15% higher than whole blood)	Hypoglycaemia may be seen following HIE, but is usually correctable with appropriate treatment. Persistently low glucose requires further evaluation.
Blood lactate	Lactate is often measured on the blood gas, and may increase rapidly to high levels following HIE, but usually falls within days and returns to normal. A persistently high lactate should trigger further investigations.
Neurophysiology	Amplitude integrated EEG (aEEG) using a cerebral function monitor and/or serial standard EEGs to identify seizures and monitor recovery of encephalopathy. Will also help diagnose neonatal epilepsy syndromes.
*Second line investigations to consider ordering which are available quickly (if concerned this is not typical HIE)*	
Urinary ketones	Urinary ketones, when present, in a neonate indicate the use of intermediary pathways of metabolism and are almost pathognomonic of the presence of a metabolic disorder.
Ammonia	In very sick neonates, ammonia, up to about 110 μmol/L may be present. Very high levels (>200 μmol/L) usually indicate a metabolic cause, for example, urea cycle defect and warrants further investigations.

HIE, hypoxic-ischaemic encephalopathy.

### Second line investigations where HIE is not confirmed

While metabolic conditions do have specific features (box 2) these overlap and gestalt diagnosis is difficult.^2^ Therefore, the diagnostic approach relies on further investigations, tailored to the clinical picture.^2–4^ We don't advise a scattergun approach to investigations. A suggested diagnostic algorithm is presented in figure 1.

**Figure 1:**
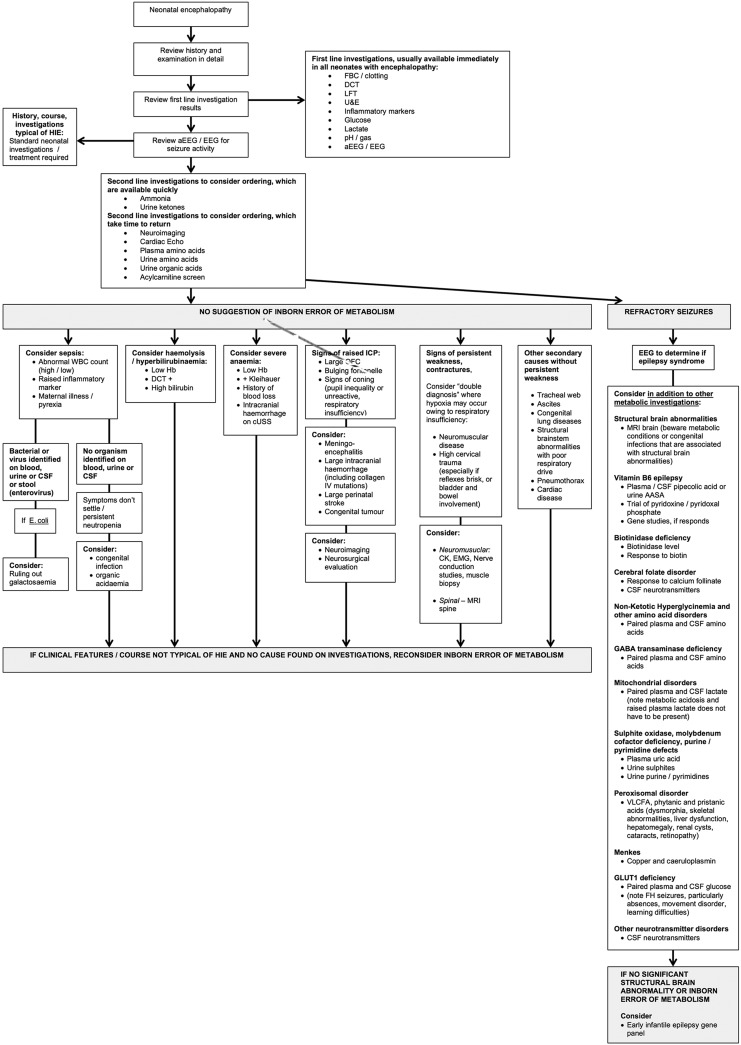
Continued

**Figure 1:**
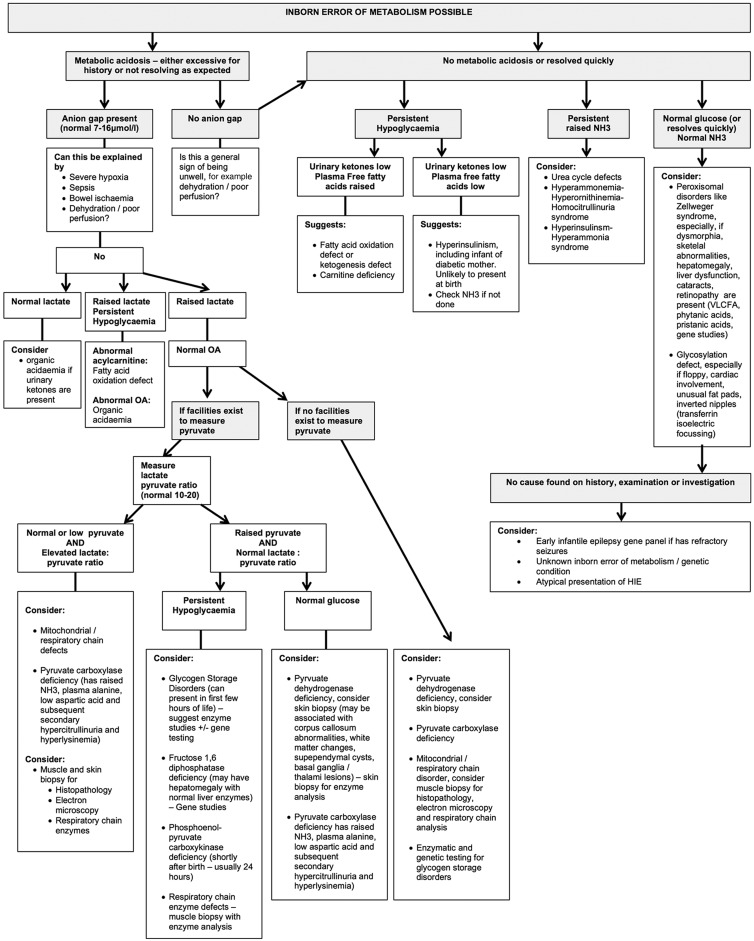
Flow chart to help to determine the cause of neonatal encephalopathy where the history and courses are not typical of hypoxia ischaemia. aAASA, alpha amino adipic semialdehyde; aEEG, amplitude integrated EEG; CK, creatine kinase; CSF, cerebrospinal fluid; cUSS, cranial ultrasound scan; DCT, direct Coombs test; EEG, electroencephalogram; EMG; electromyography; FH, family history; FBC, full blood count; GABA, Gaba-aminobutyric acid; HIE, hypoxic-ischaemic encephalopathy; LFT, liver function test; IV, intravenous; NH3, ammonia; OA, organic acids; U&E, urea and electrolytes; WBC, white blood cell count; VLCFA, very long chain fatty acids.

Features to identify include:

- Where a persistent metabolic acidosis with a raised anion gap is seen, look at the lactate:
  If the lactate has returned to normal, an organic acidaemia should be considered when urinary ketones are present.
  If the lactate is persistently high and glucose low, a fatty acid oxidation defect or organic acidaemia is possible.
  If the organic acids are normal, consider mitochondrial disease, pyruvate metabolism disorders and some of the glycogen storage disorders.
- If no persistent metabolic acidosis is noted, study the blood glucose:
  Persistent hypoglycaemia with low urinary ketones and raised plasma free fatty acids suggests a fatty acid oxidation defect or ketogenesis defect. Plasma or dried blood spot acylcarnitine profile would be of diagnostic value for fatty acid oxidation defect and various organic acidurias.
  Persistent hypoglycaemia with low urinary ketones and plasma free fatty acids suggests hyperinsulinism.
  Note that hypoglycaemia can be associated with mildly raised serum ammonia.
- Very high serum ammonia suggests urea cycle disorders or other metabolic conditions associated with secondary hyperammonaemia. Advice should be sought from the local metabolic team.
- Where no metabolic acidosis, hypoglycaemia or hyperammonaemia are found, consider a peroxisomal disorder (request very long chain fatty acids, phytanic acid and pristanic acid), or a congenital disorder of glycosylation, where inverted nipples, unusual fat pads or cerebellar involvement are seen. To diagnose the latter, the serum transferrin pattern obtained by automated isoelectric focusing should be ordered towards the end of the first month of life, as earlier samples are contaminated by mother's results.

Not all neonates with encephalopathy have seizures, but those that do have additional differential diagnoses (figure 1). These have been reviewed elsewhere.^5^ In brief, we recommend for refractory seizures:

- A trial of pyridoxine intravenously and, if no response is seen, a trial of enteral pyridoxal phosphate alongside either enteral or intravenous biotin and calcium folinate
- Investigations for the vitamin responsive epilepsies (figure 1)^5^
- Paired serum and cerebrospinal fluid (CSF) lactate to help identify mitochondrial disorders
- Paired serum and CSF amino acids to diagnose non-ketotic hyperglycinaemia and serine deficiencies
- Serum uric acid, urinary sulphites, purines and pyrimidines to diagnose molybdenum cofactor and sulphite oxidase deficiencies and purine/pyrimidine abnormalities
- Very long chain fatty acids, phytanic acid and pristanic acid to diagnose peroxisomal disorders
- Copper and caeruloplasmin to diagnose Menkes disease
- CSF neurotransmitters if cerebral folate deficiency and other neurotransmitter disorders are possible

Where no diagnosis is found, this may be an unusual presentation of HIE or an undiagnosed neurological/metabolic/genetic disorder. Where refractory seizures are present but all aetiological investigations are negative, we recommend sending DNA for analysis with an early epileptic encephalopathy gene panel or gene exome studies.

## Advances in the management of HIE

### Resuscitation

Around 85% of term babies will breathe spontaneously at birth without assistance, 10% will require stimulation, 3% will require non-invasive ventilation, 2% will be intubated and 0.1% will need chest compressions and/or adrenaline administration.^6^ It is important that skilled personal attend births, and in the case of concerning antenatal or intrapartum events, staff members with advanced neonatal resuscitation and airway skills are present. A key component in the resuscitation of the asphyxiated newborn is to establish functional residual capacity (FRC), and in doing so, enable return of spontaneous circulation (ROSC) and transition. Aeration of the previously fluid-filled lungs is necessary to reduce pulmonary vascular resistance (PVR) and increase pulmonary blood flow. Pulmonary blood flow is vital for both oxygenation and for cardiac output, as it replaces umbilical venous return as the source of preload to the left ventricle.^7^

After the cord is clamped in an apnoeic infant with sustained circulation, there is a 50% reduction in cardiac output, secondary to the sudden increase is systemic vascular resistance and the persistence of high PVR. Cardiac output is re-established with ventilation onset.^8^ For the infant who is already hypoxic, this time before establishment of FRC could exacerbate ischaemic injury. Ventilation prior to cord clamping has been shown to ameliorate swings in cardiac output and cerebral perfusion^9^ and Kluckow and Hooper^8^ propose delaying cord clamping until after ventilation onset. This would require a significant change to delivery room practice, requiring close collaboration between obstetric and neonatal staff. The current 2015 European and UK Neonatal Life Support (NLS) guidelines as well as the International Liaison Committee on Resuscitation (ILCOR) recommendation recommend delayed cord clamping of at least a minute in infants not requiring resuscitation;^6^ ^10^ further research needs to determine whether resuscitating the asphyxiated infant with the cord unclamped is of benefit. Stripping (or ‘milking’) of the cord is not recommended as a routine measure except in the context of further randomised trials.

The optimal ventilation strategy to achieve FRC and subsequently transition in the apnoeic infant is unclear. European guidelines recommend the use of 5 3-second inflation breaths, whereas American guidelines support conventional ventilation.^10^ A more prolonged (20–30 second) sustained inflation breath has been shown to hasten ROSC and transition in an asphyxiated animal model compared with conventional or ‘short’ sustained inflations.^11^ However, sustained inflation resulted in a faster and greater increase in cerebral oxygen delivery and was associated with an increase in cerebral extravasation and blood vessel disruption in asphyxiated lambs.^12^ There is therefore insufficient evidence to support the use of prolonged sustained inflations for the resuscitation of the asphyxiated infant and 5 3-second inflation breaths should be given.^10^

The concentration of oxygen used for resuscitation has been a focus of recent research. The toxicity of resuscitation in 100% oxygen is now well established. A meta-analysis of 2133 babies^13^ revealed a reduction in mortality for infants resuscitated in 21% versus 100% oxygen (relative risk (RR) 0.69, 95% CI 0.54 to 0.88) and a trend towards a reduction in HIE. In animal models of neonatal asphyxia, resuscitation with 21% versus 100% oxygen resulted in comparable or improved outcomes of death, neurobehavioural disability and cell death.^14^ In an asphyxiated lamb model, 100% versus 21% oxygen for resuscitation caused an increase in cerebral blood flow (this is counterintuitive as cerebral vasoconstriction is the typical response with oxygen).^15^ The hypoxic brain may have lost autoregulatory abilities and be ‘pressure passive’, increasing the risk of hyperoxia and flow mediated brain injury. There are no clinical studies investigating the benefit of additional oxygen commenced during resuscitation, for the infant needing extensive measures, that is, chest compressions.^6^ The current ILCOR recommendation^6^ is for resuscitation of all term infants to commence in air, and for this to be increased for the infant failing to achieve ROSC with active measures. It is suggested to reduce the oxygen content as soon as the heart rate has recovered. The concentration of oxygen to administer is unknown, and is an area for ongoing research. The European NLS guidelines suggest the use of pulse oximetry especially for deliveries where the infant is anticipated to have problems;^10^ based on normative data, the following is a guide to the acceptable preductal oxygen saturation (SpO_2_) targets during resuscitation—2 min after birth 60%, 3 min 70%, 4 min 80%, 5 min 85% and 10 min 90%.^16^

The updated 2015 European NLS guidelines suggest that attempts to aspirate meconium from the nose and mouth of the unborn infant, while the head is still on the perineum, are not recommended and initiating lung inflation within the first minute of life in non-breathing or ineffectively breathing infants should not be delayed. It is reasonable to inspect the oropharynx rapidly to remove potential obstructions but tracheal intubation should not be routine in the presence of meconium.^10^

The use of adrenaline during neonatal resuscitation is considered standard care for the infant with a heart rate <60 bpm who has failed to respond to adequate ventilation and chest compressions. Evidence for this practice is based on historical case series and extrapolated from paediatric and adult studies.^17^ A recent study in asphyxiated lambs supports the use of adrenaline in the newborn, demonstrating that chest compressions alone failed to achieve an increase in mean carotid blood flow, and that adrenaline was necessary to increase diastolic and mean carotid (and likely coronary) blood pressure, and subsequently achieve ROSC.^18^ The recommended intravenous dose for adrenaline is 10 μg/kg (0.1 mL/kg of 1:10 000 solution). If this is not effective, a dose of up to 30 μg/kg (0.3 mL/kg of 1:10 000 solution) may be tried.^10^ Endotracheal adrenaline at higher doses (50–100 μg/kg) may be used when the intravenous route is not available.^10^ ^17^ Sodium bicarbonate is not recommended during brief resuscitation. If it is used during prolonged arrests, it should be given only after adequate ventilation and circulation (with chest compressions) is established. The dose for sodium bicarbonate is between 1 mmol and 2 mmol of bicarbonate /kg (2–4 mL/kg of 4.2% bicarbonate solution). Hypoglycaemia may occur in the delivery suite and is known to exacerbate injury; glucose should be considered if there has been no response to other drugs delivered through a central venous catheter (the dose for glucose (10%) is 2.5 mL/kg (250 mg/kg)).^10^

In the past, guidelines suggested discontinuation of neonatal resuscitation at 10 min in an infant with persisting asystole despite adequate resuscitation. This was based on data from the pre-therapeutic hypothermia era showing high mortality and neurodevelopmental impairment in survivors with ROSC after 10 min. More recent publications have shown an improvement in outcome for infants with an Apgar score of 0 at 10 min, for both cooled and non-cooled infants. Rates of survival without disability were 20.5% (normothermia) to 27% (cooled) at 24 months in a recent meta-analysis^19^ and 20.8% at 6–7 years in follow-up of the NICHD cooling trial.^20^ The 2015 ILCOR consensus on science and 2015 European NLS guideline continues to support discontinuation at 10 min, although advises individualised decision making taking into consideration adequacy of resuscitation, access to cooling and parental opinion.^6^ ^10^

For infants requiring and surviving extensive resuscitation, early thought should be given to therapeutic hypothermia, discussed in further detail below. Therapeutic hypothermia is most effective when commenced as close as possible in time to the hypoxic-ischaemic event. Our practice and recommendation is to maintain normothermia (avoiding hyperthermia) until a decision to treat with cooling is made by a senior clinician. Passive cooling can then be started as soon as possible, typically in the delivery room by turning off radiant heaters. Active cooling can then be commenced in the neonatal unit. For infants born in centres not providing intensive care, infants should be promptly discussed with a tertiary neonatal unit. Cooling should be commenced as soon as possible at the referring centre, and/or by the transport service. A clinical practice guideline for cooling in transport is available.^21^ A recent clinical trial in the transport setting demonstrated that active cooling using a servo controlled device resulted in a greater number of babies achieving the target temperature than with passive cooling.^22^

The term HIE is used from this point as it is assumed other possible aetiologies of encephalopathy are excluded.

### Supportive care

Infants with HIE may have a degree of multiorgan dysfunction. The hypoxic fetus will initiate the diving reflex to preserve blood flow to vital organs, including brain, heart and adrenals, at the expense of flow to skin, splanchnic vessels, liver and kidneys.^23^ Supportive care to the infant with HIE in the neonatal intensive care unit should reflect possible hypoxic damage to the organs and be individually tailored.

Acute tubular necrosis and syndrome of inappropriate antidiuretic hormone are common, as is deranged liver function. Parenteral fluids should be restricted initially as infants will be oliguric; we would restrict fluids to 40 mL/kg/day typically until the urine output starts to increase. We administer parenteral nutrition through a central venous catheter. Trophic feeds may be started as colostrum becomes available; typically the feed volume does not increase above trophic feeds until after rewarming when the infant is less sedated. Medications requiring renal and hepatic metabolism, especially those with nephrotoxicity, should be used cautiously. Hyperglycaemia and hypoglycaemia should be avoided, as both are associated with long-term disability at 18 months or death in infants with moderate to severe HIE.^24^ Some studies suggest the particular association of hypoglycaemia with adverse outcome^25^ and the operational threshold for taking steps to raise the blood glucose is higher in infants with HIE than healthy term infants (>2.5 mmol/L vs >2.0 mmol/L). Coagulopathy due to hypoxic-ischaemic injury to the liver and bone marrow may occur. Additionally hypothermia reduces platelet aggregation, reduces enzymatic function in the coagulation cascade and can trigger disseminated intravascular coagulation. Coagulation studies should be monitored daily during cooling as laboratory evidence of coagulopathy is universal in babies with HIE, undergoing cooling. Transfusion strategies to maintain platelet counts >130×10^9^/L, fibrinogen >1.5 g/L and INR <2 may prevent clinical bleeding.^26^

Hypotension is observed in up to 62% of infants with HIE; inotropic support for HIE has been recently reviewed.^27^ Cardiac troponins I and T are established markers of myocardial ischaemia and cardiac failure in adults, children and neonates. In HIE, troponin T levels have been shown to reach a peak on day 1, remain elevated for the first week and correlate with the severity of HIE.^28^ Therapeutic hypothermia reduces cardiac output by 67% and an increase in support will usually be required doing the cooling period.^27^

Factors contributing to pulmonary dysfunction are impaired central respiratory control, pulmonary injury, persisting pulmonary hypertension (PPHN) and meconium aspiration. Adequacy of ventilation should be closely monitored and PaO_2_ and partial pressure of carbon dioxide kept within normal range. Acidosis and hypoxia should be corrected to avoid additional brain injury and PPHN; hyperoxia and hypocarbia should be avoided as they are detrimental to long-term outcome^29^ ^30^

### Seizure management

Seizures are a common feature of HIE; however, it should be noted that around 34% of neonatal seizures have clinical features that can be seen, and only 27% of those were correctly identified by neonatal staff. In addition, over 70% of what are thought to be seizures are not associated with epileptiform discharges on electroencephalogram (EEG), highlighting the importance of neurophysiological monitoring.^31^ In HIE, seizures usually occur on day 1, and those seen prior to 6 hours of age should raise suspicion of earlier in utero insult. Seizures increase cerebral metabolic demand, trigger release of excitatory neurotransmitter and cause cardiorespiratory instability, all exacerbating neuronal injury. Increased seizure burden has been significantly independently associated with more severe injury on MRI (OR 5.00, 95% CI 1.47 to 17.05, p=0.01)^32^ and with poorer scores on neurodevelopmental follow-up at 18 months.^33^ Therapeutic hypothermia reduces seizure burden in infants with mild–moderate, but not severe HIE.^34^ A rebound increase in seizures can be seen with rewarming.^32^

There are no universal guidelines for the management of seizures in neonatal HIE with strategies differing between centres. Status epilepticus and frequent seizures should be treated, although there is debate about whether to treat less frequent seizures, or electrographic only (ie, not clinically apparent) seizures. In one study treatment of electrographic seizures using amplitude integrated EEG (aEEG), compared with treatment of clinical seizures (aEEG concealed) was associated with lower seizure burden and less severe MRI injury rating scores. However there was no difference in developmental outcome at 18 months.^33^

The first line anticonvulsant drug is phenobarbitone,^35^ although this will only control seizures in 50% of neonates.^36^ Common second line agents include phenytoin, benzodiazepines, and in Europe, lidocaine. Given the potential toxicity and limited efficacy of these traditionally used drugs, new agents are being sought. There is limited pharmacokinetic, efficacy, toxicity and dosing information available for the newer anticonvulsants (ie, levetiracetam and topiramate) in neonates—a recent review is available.^37^ Levetiracetam has attractive characteristics (ie, CYPP450 independence, intravenous formulation available, 100% oral bioavailability, no drug interactions and no protein binding) and is already in off-label use in some centres. It is important to establish safety and efficacy, as evidenced by a recent trial into a similarly promising agent, bumetanide, which showed poor efficacy and an increase in hearing loss.^38^ A Cochrane review demonstrated that there is no evidence to support the use of prophylactic anticonvulsants after perinatal asphyxia.^39^ Anticonvulsants are usually only required in the first week because seizures are ‘acute symptomatic’ and burn out with time. Occasionally longer-term therapy is required in severely affected infants.

## Encephalopathy assessment and prognostication

A rapid clinical assessment will be required to determine eligibility for therapeutic hypothermia within the first 6 hours of life. Following this, regular reassessment and investigation is prudent to determine progression of encephalopathy, exclude other causes of encephalopathy and provide prognostic information to families. A current review of the prognostic value of clinical assessment and various investigations in HIE is available.^40^

### Neurophysiology

EEG and aEEG are important tools for assessment of severity of HIE, monitoring improvement over time and for recognition of seizures. Both have advantages and disadvantages. aEEG is readily available at all times of the day on the neonatal intensive care unit, can demonstrate background abnormalities and sleep wake cycling, and is interpretable at the bedside. aEEG can also detect a third of single seizures and two-thirds of repetitive seizures, but those that are short lasting (<30 s) or distant from the electrodes may be missed.^41^ Nevertheless, aEEG is clearly superior to clinical detection of seizures alone.^42^

Multichannel EEG is the gold standard, however technicians are required to site EEG leads and may not be available at all times of the day. Specialised neurophysiological interpretation and prompt reporting of the EEG are also required and these resources may not be available in all hospitals. Abnormalities of background EEG pattern and the loss of sleep wake cycling are commonly early after hypoxia ischaemia, and can be used to assess clinical recovery and predict outcome. With therapeutic hypothermia, the optimal time to assess aEEG for prognosis is 48 hours, with the return to a discontinuous normal voltage, or a continuous normal voltage being associated with good outcome, particularly if sleep wake cycling is present.^43^ A recent meta-analysis of aEEG background and prediction of outcome is available.^44^

### Cranial ultrasound

Cranial ultrasound (CrUSS) is a simple, non-invasive and convenient initial imaging assessment for infants with HIE. Cerebral oedema may be evident, with sparkly echo reflectance of the parenchyma, obscuration of the sulcal markings and closure of the fissures. Slit-like ventricles are a normal finding in term infants. In severe HIE there is increased echogenicity in the thalamus and basal ganglia. However CrUSS is a poor prognostic indicator, with only a 79% (95% CI 37% to 97%) sensitivity and 55% (95% CI 35% to 70%) specificity for abnormal outcome.^40^ Cerebral flow velocity can also be measured using Doppler studies. In healthy term infants in the first 24 hours, the average resistance index (RI) is 0.726 (SD 0.057). A reduction in RI to ≤0.55 is associated with poor outcome after perinatal asphyxia, although with cooling the positive predictive value falls from 84% to only 60% (95% CI 45% to 74%).^45^

### MRI and magnetic resonance spectroscopy (MRS)

MRI is the imaging modality of choice for assessment of injury severity and prognostication in NE and a recent framework for practice outlines the clinical indications, acquisitions and reporting for neonatal and fetal MRI.^46^ Changes on MRI scanning in the neonatal period are reflective of pattern of injury (basal ganglia predominant in ‘acute-total’, watershed predominant in ‘prolonged-partial’ or widespread injury in ‘severe-global’) and correlate well with pattern of neurodevelopmental impairment.^47^ MRI findings will change with time, and early scans may miss the full extent of injury. The practice parameter concludes that imaging should include conventional structural T1 weighted and T2 weighted images, diffusion weighted images, and, where available, single-voxel MRS and be performed between 5 days and 14 days.^46^

Injury on conventional MRI (T1 and T2) within the first 2 weeks of life is 98% (95% CI 80% to 100%) sensitive and 76% (95% CI 36% to 94%) specific for the prediction of long-term outcome. Diffusion weighted imaging and the apparent diffusion coefficient may demonstrate abnormalities earlier than conventional MRI, but they are of less prognostic value (sensitivity 58% (95% CI 24% to 84%), specificity 89% (95% CI 62% to 98%); and sensitivity 79% (95% CI 50% to 93%), specificity 85% (95% CI 75 to 91) respectively).^40^

MRS is increasingly used as a quantitative tool both for clinical and research prognostication. ^1^H MRS can be used to measure peaks of N-acetylasparate (NAA), choline, creatine, lactate and the relative ratios of each in the thalamus and basal ganglia. In Thayyil *et al*'s^48^ meta-analysis, lactate/NAA >0.29 (0.24 to 0.4) had a sensitivity of 0.82 (95% CI 0.74 to 0.89) and a specificity of 0.95 (95% CI 0.88 to 0.99) for predicting an abnormal outcome. On further review of studies that measured both MRS and conventional MRI, Lac/NAA was more specific (98% (95% CI 87% to 100%) vs 76% (95% CI 61% to 88%)) and equally as sensitive (86% (95% CI 72% to 95%) vs 80% (95% CI 65% to 90%) as conventional MRI for prediction of long-term outcome.^48^

Therapeutic hypothermia significantly reduced the number of infants with abnormal MRI findings with a similar predictive accuracy of abnormal MRI (day 8) for outcome in the TOBY trial at 18 months for cooled and non-cooled infants.^49^ Recent studies however suggest that following cooling a ‘normal’ MRI may not always predict normal outcome accurately—Rollins *et al*^50^ describe a negative predictive value of a normal MRI of 74% in their series.

### Near-infrared spectroscopy

Near-infrared spectroscopy can be used as a non-invasive bedside tool to obtain real time information on changes in cerebral oxygenation and haemodynamics. Regional cerebral oxygenation (SctO_2_) and cerebral blood volume are higher on day 1 in infants with HIE with adverse outcomes compared with those with a favourable outcome.^51^ ^52^ Fractional tissue oxygen extraction remains stable in infants with normal outcome but decreases after 24 hours in infants with adverse outcome.^44^ Wavelet coherence analysis has been used to assess the dynamic status of cerebral autoregulation during therapeutic hypothermia in HIE. Based on this method, significant in-phase and antiphase coherence between spontaneous oscillations in mean arterial pressure and SctO_2_ were found; both appeared to be related to worse clinical outcomes.^53^ These findings support the feasibility of using this method to assess cerebral autoregulation in neonates with HIE as well as using this as a short-term and long-term outcome measure. Broadband near-infrared spectroscopy (NIRS) measures concentration changes of the cytochrome c oxidase (oxCCO) redox state. CCO is the terminal electron acceptor within mitochondrial electron transport chain and is responsible for >95% of ATP synthesis; preclinical studies suggest a correlation between CCO and nucleoside triphosphate/phosphate pool recovery after hypoxia ischaemia.^54^

### Prechtl

Prechtl's method on the qualitative method of general movements (GMsA) is non-invasive observational assessment performed using video or direct inspection while the infant is in quiet wakefulness. General movements (GMs) are whole body movements believed to be important for the development of voluntary motor pathways, and normally progress in two predictable developmental stages. ‘Writhing movements’ are low-moderate speed fluid movements of the trunk and limbs, and occur up to 6–9 weeks. ‘Fidgety movements’, are most evident at 12 weeks. Abnormalities of writhing movements (poor repertoire or cramped synchronised) and fidgety movements (absent or abnormal) predict neurodevelopmental outcome.^55^ In 259 high-risk (preterm and NE term) infants, absent fidgety movements at 12 weeks had 98% sensitivity and 94% specificity for CP at 1 year.^56^ In smaller cohorts of term asphyxia infants only, results are similar^57^ with abnormalities on GMsA highly correlating with MRI abnormalities.^58^

### Blood biomarkers

A key to improving outcome is the identification of early biomarkers of brain injury that can be used to direct interventions, gauge treatment effects and provide prognostic information for parental counselling. There is no serum biomarker in current clinical use for NE, however. Various biomarkers of brain injury in blood, urine and CSF have been proposed, including S100 calcium-binding protein B (S100B), glial fibrillary acidic protein (GFAP), ubiquitin carboxyl-terminal hydrolase L1 (UCH-L1), creatine kinase brain band, neuron-specific enolase (NSE), malondialdehyde and proinflammatory cytokines. Massaro *et al* have shown that elevated serum S100B and NSE levels measured during hypothermia are associated with neuroradiographic and clinical evidence of brain injury in NE.^59^ Chalak *et al*^60^ were able to stratify HIE into mild, moderate and severe based on cord blood GFAP and ubiquitin carboxy-terminal hydrolase L1. These brain-specific proteins may be useful immediate biomarkers of cerebral injury severity but still need to be independently validated in large cohorts before they are ready for clinical implementation in practice.

## Neuroprotective therapy in HIE

### Therapeutic hypothermia

The key principle to postnatal therapeutic interventions is the concept of delayed secondary injury. Following birth and resuscitation, the neonatal brain has a period of partial recovery, followed by a latent phase lasting 1–6 hours. In moderate to severe encephalopathy the brain then enters a phase of secondary injury with near complete mitochondrial energy production failure, cytotoxic oedema, cell death and clinical deterioration often with seizures. This occurs for approximately 6–15 hours following the hypoxic-ischaemic event.^61^ The latent phase provides a therapeutic window during which therapy can be provided to prevent secondary injury.

Therapeutic hypothermia commenced during the latent phase has been the most important recent innovation in the care of HIE. Therapeutic hypothermia improves outcomes of death and disability.^62^ ^63^ Longer-term developmental outcomes are emerging and support findings at 18–24 months.^64^ ^65^ Eligibility criteria for cooling differ slightly between the RCTs and now between neonatal units. However, the overall principals are the same—there should be evidence of recent intrapartum asphyxia for the term or near-term infant and the infant should demonstrate encephalopathy. Our practice is to use the TOBY trial eligibility criteria.^66^

It is clear from available clinical and preclinical evidence that moderate therapeutic hypothermia should be implemented as soon as possible, before onset of secondary injury and continued until this period of secondary energy failure has resolved.^61^ Cooling should be started as soon as possible. Infants cooled within 3 hours of birth have better neurodevelopmental outcomes compared with infants whose cooling commences between 3 hours and 6 hours.^67^ Following 72 hours of cooling, infants should be slowly rewarmed (0.5°/hour). This is based on animal data showing increased seizures^68^ and increased cortical apoptosis^69^ with rapid rewarming. Longer or deeper cooling to <33.5° and/or for >72 hours has not been shown to be of benefit, and is harmful.^70^ ^71^

### Future neuroprotective adjuncts

The number needed to treat with therapeutic hypothermia for an additional beneficial outcome is 7 (95% CI 5 to 10) from 8 studies, 1344 infants.^62^ Importantly, this means there is still a large number of infants for whom this therapy is ineffective. Adjuvant therapy to hypothermia is a current focus of research and has been reviewed in more detail elsewhere.^72^ ^73^ Some of the more promising neuroprotective agents, scored by an international group of neuroscientists^81^ include melatonin, erythropoietin, inhaled xenon and argon, allopurinol, stem cells, cannabinoids and magnesium (table 2).

**Table 2:** Summary of preclinical and clinical trial studies on seven promising adjunct neuroprotective agents

Adjunct therapy	Mode of action	Examples of recent preclinical trials	Clinical RCTs
Melatonin	Endogenous hormone which entrains the circadian rhythm at physiological doses. At high pharmacological doses melatonin is a powerful antioxidant and antiapoptotic agent.	Systematic review and meta-analysis of 400 adult rodents showed a 43% reduction in stroke infarct size with melatonin.^74^ A piglet study showed augmentation of brain protection with high dose melatonin at 10 min and cooling versus cooling alone.^75^	Oral melatonin (10 mg/kg/day 5 doses) tablets crushed in 5 mL distilled water. n=15 cooled, n=15 cooled plus melatonin, n=15 controls.^76^
Erythropoietin (Epo)	*Acute actions*: neurotrophic, anti-inflammatory, antiapoptotic, antioxidant *Chronic actions*: erythropoiesis, angiogenesis, oligodendrogenesis, neurogenesis.	Non-human primate model—hypothermia+Epo treatment improved outcomes in non-human primates exposed to umbilical cord occlusion.^77^	NEAT trial—safety and PK.^78^ Phase II trial of hypothermia and Epo showed less MRI injury and better short-term outcome.^79^ Phase III trial is now underway in the USA.
Xenon	Inhibits NMDA signalling, antiapoptotic.	Preclinical piglet studies showed benefit of combined cooling and xenon compared with no treatment.^80^ ^81^	No evidence of short-term benefit with xenon and cooling above cooling alone, using MRS lactate/NAA as a surrogate outcome.^82^
Argon	GABA agonist and oxygen type properties. Antiapoptotic.	Preclinical piglet study showed brain protection on MRS and histology with 50% argon and cooling compared with cooling alone.^83^	Phase II trials pending regulatory approval.
Allopurinol	Reduces free radical production and in high doses acts as a free radical scavenger and free iron chelator.	Improved ^31^P MRS metabolites and MRI values with allopurinol in piglets.^84^	ALBINO trial to start in Europe 2017—to assess benefit of early allopurinol at 30 min plus cooling versus cooling alone.
Stem cells	Paracrine signalling—not cellular integration or direct proliferative effects.	Evidence of improved neurological outcome and reduced histological injury.^85^	Autologous umbilical cord cells in HIE demonstrated feasibility.^86^
Magnesium	Prevention of excitatory injury by stabilisation of neuronal membranes and blockade of excitatory neurotransmitters, for example, glutamate.	Magnesium alone has not been protective in piglet models of hypoxia.^87^ Combinations of magnesium with cooling has shown benefit.^88^	Recent meta-analysis shows no evidence of benefit.^88^ A multicentre pilot RCT reported safety but no outcome data, larger RCT to follow^89^

HIE, hypoxic-ischaemic encephalopathy; GABA, gamma-aminobutyric acid; MRS, magnetic resonance spectroscopy; NAA, N-acetylasparate; NMDA, N-methyl-D-aspartate; PK, pharmacokinetics; RCT, randomised controlled trials.

## Follow-up

A review on the follow-up of survivors of term HIE discusses the clinical and imaging diagnostic criteria for HIE, which are essential to decisions about follow-up.^90^ The recommendations for follow-up and intervention are based on the clinical condition of the infant at the time of discharge from intensive care, including an assessment of feeding, vision, hearing and whether seizures continue to be present. Although the number of survivors from HIE is lower than the number of survivors of extreme prematurity, the proportion of neonates with long-term sequelae is higher. All neonates with Sarnat stages 2 (moderate) and 3 (severe) should be enrolled in follow-up programmes. Early assessments (at 4–8 months) focus on head growth, general health and motor neurodevelopment. Assessments at 12–24 months focus on cognitive skills and language development. Preschool assessments are also strongly recommended to provide for the identification of children requiring early education programmes.

## Documentation and medicolegal matters

Approximately 13% of infants with NE will go on to develop CP. In the USA between 1985 and 2008, the brain-damaged infant was the leading type of paediatric medicolegal claim.^91^ In the UK between 2012 and 2013, health trusts spent £482 million on ‘maternity negligence’, a fifth of the total maternity health expenditure.^92^ While well recognised that the obstetrician may be liable for the death or long-term disability supposedly arising from negligence at the time of birth, increasingly, the paediatrician is being taken to court. Reasons for malpractice claims include, but are not limited to, substandard resuscitation technique, failure to transfer an infant to a neonatal unit in a timely manner and failure to refer for, or initiate therapeutic hypothermia in a timely manner.^91^

The clinician must fully document the resuscitation of infants with potential HIE. This should include time of arrival, a thorough description of the infant’s clinical status as this evolves during the first minutes and hours of life, time of each resuscitative intervention, cord and early blood gas parameters and most importantly decisions made re therapeutic hypothermia. It should be clearly stated why the infant is eligible or ineligible for cooling, and at what time cooling commenced.

## Redirection of care and organ donation

Mortality rates in moderate to severe HIE treated with therapeutic hypothermia, are ∼25%.^62^ Most deaths occur after redirection of care, when clinical evidence supports brain death or devastating neurological injury. Guidelines for the diagnosis of brain death in the neonate are available;^93^ ^94^,they are, however, not often used. More commonly, clinicians and families agree to cease life-sustaining therapy when clinical assessment supports severe neurological injury—persisting encephalopathy and low voltage aEEG after rewarming±severe injury on neuroimaging.

Organ donation, from severely affected HIE donors, is becoming a possibility. In the USA, neonatal organ donation is more established, while in the UK it is gaining momentum.^95^ In one US cohort, profound central nervous system injury and/or encephalopathy was the most common cause of death for potentially eligible neonatal donation after cardiac death donors.^96^ Importantly, despite the global hypoxia ischaemia suffered in HIE, organs such as the kidney usually recover once transplanted. Eligibility criteria for donation and organs harvested differ between transplant centres. Commonly there are weight restrictions (ie, >2 kg) and potential donor organs depend on whether harvested before (ie, after brain death) or after circulatory death. Most cases of neonatal solid organ donation to date are of en bloc kidneys to an adult donor, harvested after confirmation of circulatory death—as was the case for the first neonatal organ donation in the UK in 2013.^97^ In the USA a neonate with severe HIE donated her heart, liver and kidneys after determination of brain death.^98^ To our knowledge, this has yet to be performed in the UK, due, until recently, to the lack of guidelines for determining brain death in the neonate. A guideline for diagnosing brain death in infants less than 2 months has recently been published by the UK Royal College of Paediatrics and Child Health.^94^ Other impediments to neonatal organ donation include a lack of awareness of available services and a reluctance of staff to discuss donation with grieving families. Families are increasingly aware of organ donation, and may be the first to raise the subject. Our advice is to always consider organ donation, discuss with your local transplant service as soon as possible prior to redirection of care, and offer the choice to the families of eligible infants.

## The next 10 years

The challenge over the next 5–10 years will be to assess (in phase I and II trials) which adjunct therapy or combination is safe and optimises hypothermic brain protection. Optimal care may require tailoring treatments according to gender, genetic risk, injury severity and inflammatory status. Early biomarkers, once validated, will allow infants to be stratified and treatment effects to be assessed. Rescue treatment may be needed in some infants not responding to cooling. Until now, treatments have been targeted to the early acute phase of injury; enhancing repair and neurogenesis during the tertiary phase will require collaboration between neonatologists and paediatric neurologists.
